# Huangkui Capsule Attenuates Lipopolysaccharide-Induced Acute Lung Injury and Macrophage Activation by Suppressing Inflammation and Oxidative Stress in Mice

**DOI:** 10.1155/2021/6626483

**Published:** 2021-09-22

**Authors:** Jinfang Deng, Zhenpeng He, Xiuru Li, Wei Chen, Ziwen Yu, Ting Qi, Shuangyi Xu, Zhengxin Xu, Lei Fang

**Affiliations:** ^1^Department of Pathlogy, First Medical Center of PLA General Hospital, Beijing 100853, China; ^2^Institute of Translational Medicine, Medical College, Yangzhou University, Yangzhou 225009, China; ^3^Jiangsu Key Laboratory of Experimental & Translational Non-Coding RNA Research, Yangzhou University Medical College, Yangzhou 225009, China

## Abstract

**Background:**

Huangkui capsule (HKC) comprises the total flavonoid extract of flowers of *Abelmoschus manihot* (L.) Medicus. This study aimed to explore the effects of HKC on lipopolysaccharide- (LPS-) induced acute lung injury (ALI) in mice and LPS-stimulated RAW 264.7 cells.

**Methods:**

Enzyme-linked immunosorbent assay, histopathology, spectrophotometry, and quantitative real-time polymerase chain reaction were used for the assessments. Statistical analysis was performed using a one-way analysis of variance.

**Results:**

LPS significantly increased lung inflammation, neutrophil infiltration, and oxidative stress and downregulated lung miR-451 expression. Treatment with HKC dramatically, reduced the total cell count in the bronchoalveolar lavage fluid (BALF), and inhibited myeloperoxidase activity in the lung tissues 24 h after LPS challenge. Histopathological analysis demonstrated that HKC attenuated LPS-induced tissue oedema and neutrophil infiltration in the lung tissues. Additionally, the concentrations of tumour necrosis factor- (TNF-) *α* and interleukin- (IL-) 6 in BALF and IL-6 in the plasma reduced after HKC administration. Moreover, HKC could enhance glutathione peroxidase and catalase activities and upregulate the expression of miR-451 in the lung tissues. In vitro experiments revealed that HKC inhibited the production of nitric oxide, TNF-*α*, and IL-6 in LPS-induced RAW 264.7 cells and mouse primary peritoneal macrophages. Additionally, HKC downregulated LPS-induced transcription of TNF*-α* and IL-6 in RAW 264.7 cells.

**Conclusions:**

These findings suggest that HKC has anti-inflammatory and antioxidative effects that may protect mice against LPS-induced ALI and macrophage activation.

## 1. Introduction

Acute lung injury (ALI), a common lung inflammatory disease, is characterised by tachypnoea, progressive hypoxaemia, and reduced lung compliance. Acute respiratory distress syndrome (ARDS), a more severe manifestation of ALI, even results in respiratory failure with a mortality of approximately 40% [1]. The lung-protective mechanical ventilation is a standard therapeutic method for ALI/ARDS patients. However, mechanical ventilation itself can lead to biotrauma, increasing lung inflammation and worsening clinical condition. Additionally, use of corticosteroids to attenuate inflammation remains limited for timing and duration of administration. ALI/ARDS patients may benefit from corticosteroids upon initiation of the therapy at early stages of the syndrome; however, its administration after 14 days of disease onset is not favourable [2]. Despite the wide use of these supportive clinical approaches in the last decades, the survival of adult patients with ARDS has not improved [3]. Therefore, it is necessary to find a new and effective therapeutic strategy for the treatment of ALI.

Pulmonary bacterial infection is a leading cause of ALI [4]. Lipopolysaccharide (LPS), a major component of the Gram-negative bacterial cell wall, induces the innate immune response by activating macrophages [5]. The macrophage-derived cytokines, including tumour necrosis factor-*α* (TNF-*α*) and interleukin- (IL-) 6, have been considered as important regulators in the development of inflammation. The excess of inflammatory mediators contributes to further neutrophil infiltration into the lung tissues, damages the alveolar-capillary barrier, and ultimately impairs gas exchange. High levels of TNF-*α* and IL-6 in the bronchoalveolar lavage fluid (BALF) of ALI/ARDS patients is associated with poor prognosis. The degree of neutrophil activation increases with the degree of lung injury and the levels of TNF-*α*, IL-6, and IL-8 [6]. Moreover, intense inflammation results in the overproduction of reactive oxygen species (ROS) from phagocytic leukocytes. ROS production in turn leads to the opening of interendothelial junctions and promotes the migration of inflammatory cells across the endothelial barrier into the tissues [7]. Antioxidant therapy as a complementary measure improves oxygenation rates and glutathione levels and strengthens the immune response, indicating a method to bypass the excessive inflammation associated with the high oxidation state existing in ALI/ARDS patients [8]. Thus, the blockage of inflammation and oxidative stress in the lung might be a potential therapeutic strategy against ALI.

*Abelmoschus manihot* (L.) Medicus (syn.: Hibiscus manihot), listed as Huang Shu Kui Hua (in Chinese) in Chinese Pharmacopoeia, has been used to treat inflammation, pain, urinary infections, and chronic bronchitis as a folkloric medicinal plant [9]. Huangkui capsule (HKC), a commercial product, contains the total flavonoid extract of the flowers of *A. manihot*. The principal bioactive components of HKC, including isoquercitrin, hibifolin, myricetin, quercetin-3′-O-D-glucoside, quercetin, hyperoside, rutin, and gossypetin, have been identified previously [10, 11]. Clinically, HKC has been used to treat chronic glomerulonephritis, diabetic nephropathy, and nephrotic syndrome [11–13] for two decades. Some pharmacological studies found that the effects of HKC might be associated with its antioxidant and anti-inflammatory properties. In the adriamycin-induced nephropathy rats, HKC could ameliorate renal inflammation by reducing the infiltration of ED^1+^ and ED^3+^ macrophages in glomeruli and TNF-*α* levels in the kidney [13]. The *A. manihot* extract protected adriamycin-induced tubule injury via inhibition of ROS-ERK1/2-NLRP3 inflammasomes [14]. Additionally, total flavones of *A. manihot* have therapeutic effects on other inflammation-related diseases or oxidative injuries (e.g., Crohn's disease [15], poststroke depression injury [16], and myocardial ischaemia/reperfusion damage [17]). Recently, HKC has been used to treat airway inflammation in allergic asthma patients in China. However, the protective effect of HKC in neutrophil-dominant lung injury has not been revealed yet. In this study, we explored whether HKC exerts anti-inflammatory and antioxidant activities in LPS-induced ALI in mice and LPS-stimulated RAW 264.7 cells.

## 2. Methods

### 2.1. Mice and Cell Line

C57BL/6J mice (male, 6–8 weeks old, 18–20 g) were purchased from the Comparative Medicine Centre of Yangzhou University (license number: 2017–0044). The murine macrophage RAW 264.7 cell line was purchased from the Cell Bank of the Chinese Academy of Science (Shanghai, China).

### 2.2. Drugs and Reagents

HKC was purchased from Jiangsu Suzhong Pharmaceutical Group Co., Ltd. (Taizhou, Jiangsu, China, batch number: 19101911). One capsule of HKC contains 500 mg extract of *A. manihot* flowers. Enzyme-linked immunosorbent assay (ELISA) kits for mouse TNF-*α* and IL-6 were obtained from Biolegend Co. (San Diego, California, USA). The EL-TMB Chromogenic Reagent Kit and 3, 3′, 5, 5′-tetramethylbenzidine (TMB) were purchased from Sangon Biotech Co. (Shanghai, China). Dexamethasone sodium phosphate (DEX) was purchased from Shenyang Guangda Pharmaceutical Co. (Shenyang, Liaoning, China). The glutathione peroxidase (GPx) assay kit was purchased from Nanjing Jiancheng Bioengineering Institute (Nanjing, China). LPS, TRIzol, Enhanced Cell Counting Kit-8, and dichloro-dihydro-fluorescein diacetate (DCFH-DA) were obtained from Beyotime Biotechnology (Haimen, Jiangsu, China). PrimeScript RT MasterMix was purchased from Takara Biotechnology (Dalian) Co., Ltd. (Dalian, Liaoning, China). HiScript III RT SuperMix for qPCR was obtained from Vazyme Biotech Co., Ltd. (Nanjing, Jiangsu, China). QuantiNova SYBR Green PCR Kit was purchased from Qiagen Co. (Hilden, Germany). All other reagents were of analytical grade.

### 2.3. LPS-Induced ALI Mice and HKC Administration

Mice were allowed to acclimate for one week and were divided randomly into six groups: control, LPS, LPS + HKC (150, 300, and 600 mg/kg), and LPS + DEX (5 mg/kg; anti-inflammatory positive control). Mice (4–5 mice/cage) were kept in standard laboratory conditions of temperature (22 ± 2°C) and a 12 h light/dark cycle.

**Figure 1:**
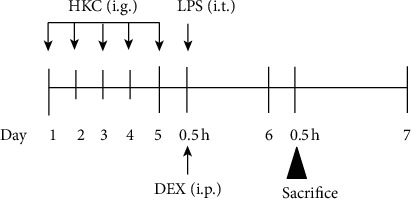
The experimental protocol. Schedule of pretreatment with HKC and positive drugs in mice induced by LPS. Mice were intragastrically administered HKC once daily for 5 days. DEX (5 mg/kg) was intraperitoneally injected once 30 min before LPS instillation.

The experimental protocol is shown in Figure 1. HKC was dissolved in 0.5% sodium carboxymethyl cellulose to a concentration of 50 mg/mL and was administered intragastrically once per day for 5 consecutive days. DEX was administered intraperitoneally once before LPS instillation. Mice in the control and model groups received equal volume of sterile 0.9% saline. At 0.5 h after drug administration on day 5, mice in the model, DEX, and HKC groups were anaesthetised with intraperitoneal administration of pentobarbital sodium (50 mg/kg) and were subsequently administered intratracheal LPS (250 *μ*g/kg) instillation. Mice in the control group were provided equal volume of saline instead of LPS. At 24 h after LPS challenge, the blood, BALF, and lung tissues were collected for experiments. Animal experiments were approved by the Animal Ethics Committee of the Medical College of Yangzhou University (YXYLL-2020-136).

### 2.4. IL-6 Levels in Plasma

At 24 h after LPS challenge, mice were anaesthetised by inhalation of diethyl ether, and their blood was collected via retroorbital puncture using heparinised capillary tubes. Subsequently, blood samples were centrifuged (1100 × g, 10 min) at 4°C to obtain plasma samples. The plasma level of IL-6 was assayed using ELISA kits according to the manufacturer's instructions.

### 2.5. TNF-*α* and IL-6 Levels and Total Cell Count in BALF

BALF was collected as described previously [18]. The lungs were lavaged three times via intratracheal intubation with cold phosphate-buffered saline (PBS) to collect approximately 1 mL of BALF. The total cell count was determined using a haemocytometer. Subsequently, BALF was centrifuged at 500 × g for 10 min at 4°C, and the levels of TNF-*α* and IL-6 in supernatants were measured through ELISA. Thereafter, the cell pellet was resuspended in 500 *μ*L of PBS. The cell suspension was diluted (1 : 5) with PBS to prepare a smear using the cytospin (Thermo Scientific, USA). The number of neutrophils was counted through haematoxylin and eosin (H&E) staining.

### 2.6. Histopathological Examination of Lung Tissues

The upper right lobes were fixed in 10% formalin solution for 24 h and then subjected to a standard histological procedure. Lung sections of 4 *μ*m thickness were subjected to H&E staining and were observed under a light microscope (Nikon Eclipse 80i, Japan). A semiquantitative score method was used to assess the degree of lung injury, as previously described with some modifications [19]. Each specimen was evaluated based on parameters such as the degree of alveolar congestion, haemorrhage, and oedema, and degree of neutrophil infiltration in the airspace or interstitium. Scores of each parameter ranged from 0 to 4 (0, minimal; 1, mild; 2, slight; 3, moderate, and 4, severe). The total score (0–12) was obtained by summing the score of each parameter.

### 2.7. Myeloperoxidase (MPO) Activity Assay

The lower right lobes were weighed and homogenised in 0.5% cetyltrimethyl ammonium bromide. After the homogenate was centrifuged (12,000 × g, 15 min) at 4°C, the supernatant was collected and assayed for MPO activity, according to our previously reported method [18]. MPO activity in the lung tissues was expressed as units per milligram of tissue.

### 2.8. Catalase (CAT) and GPx Activities in Lung Tissues

Left lung samples from mice were homogenised in PBS, and the GPx activity was measured according to the manufacturer's instructions. Lung CAT activity was detected according to our previously reported method [18]. Protein concentration in the tissue homogenate was measured using the BCA kit. CAT and superoxide dismutase activities in the lung tissues were expressed as units per milligram of protein.

### 2.9. Nitric Oxide, TNF-*α*, IL-6, and ROS Production in LPS-Stimulated Macrophages

RAW 264.7 cells were cultured in complete Dulbecco's modified eagle's medium at 37°C with 5% CO_2_. Cells were seeded into 96-well plates at a density of 2 × 10^5^ cells per well and were incubated with HKC (25, 50, 100, and 200 *μ*g/mL). After 24 h, cell viability was assessed using a CCK-8 kit.

Similarly, cells were seeded in 96-well plates and incubated with HKC (25, 50, 100, and 200 *μ*g/mL) and LPS (100 ng/mL). After 24 h of stimulation by LPS, the supernatant was collected to assess nitric oxide release and cytokine levels. Nitric oxide levels were measured by adding Griess reagent, and optical densities were assessed using a microplate reader (Thermo Fisher, USA) at a wavelength of 492 nm. The concentrations of TNF-*α* and IL-6 were measured using ELISA. The intracellular ROS assay was performed using DCFH-DA. Macrophages were incubated with DCFH-DA (10 *μ*mol/L) at 37°C for 30 min in the dark after washing with PBS twice, and DCF fluorescence intensities were measured using a microplate reader at the excitation and emission wavelengths of 485 and 535 nm, respectively.

Mouse primary peritoneal macrophages were collected from male C57BL/6J mice 4 days after intraperitoneal injection of 1 mL of 4% thioglycollate medium, as previously described [20], with some modifications. The cells were seeded into 96-well plates and were incubated overnight to allow for attachment to the plate. The medium was then replaced with fresh medium, and the cells were incubated with LPS and HKC. After 24 h of stimulation by LPS, the supernatants were collected for nitric oxide release and cytokine assays.

### 2.10. Quantitative Real-Time Polymerase Chain Reaction (PCR)

Total RNA was extracted from the lung tissues or RAW 264.7 cells using TRIzol reagent, according to the manufacturer's instructions. The cDNA was obtained using the reverse transcription kit. Quantitative real-time PCR was performed using the ABI 7500 Real-Time PCR system (Applied Biosystems, Foster City, CA, USA). The relative expression of miR-451, TNF*-α*, and IL-6 was calculated using the 2^−ΔΔCt^ formula and was normalised to that of GAPDH (mRNA) or U6 (miRNA). The following primers were used: miR-451, forward 5ʹ-AAA CCG TTA CCA TTA CTG AGT T-3ʹ, and the universal adaptor PCR primer was used as the reverse primer; TNF*-α*, forward 5ʹ-GCC TAT GTC TCA GCC TCT T-3ʹ and reverse 5ʹ-GGT TGA CTT TCT CCT GGT AT-3ʹ; IL*-6*, forward 5ʹ-CGA TAG TCA ATT CCA GAA ACC GC-3ʹ and reverse 5ʹ-TTG GGA GTG GTA TCC TCT GTG AAG-3ʹ; and GAPDH, forward 5ʹ-CAA AAT GGT GAA GGT CGG TGT G and reverse 5ʹ- TGA TGT TAG TGG GGT CTC GCT C.

### 2.11. Statistical Analysis

Prism 8.0 (GraphPad Software, San Diego, CA, USA) was used to perform statistical analysis. Normally distributed data were expressed as means ± standard deviations and analysed using a one-way analysis of variance followed by Tukey's test. For lung injury scores, data were expressed as medians (ranges). The Kruskal–Wallis test and Dunn's test were used. Differences were considered to be significant at *P* < 0.05.

## 3. Results

### 3.1. HKC Suppressed the Inflammatory Response in Lung Tissues

**Figure 2:**
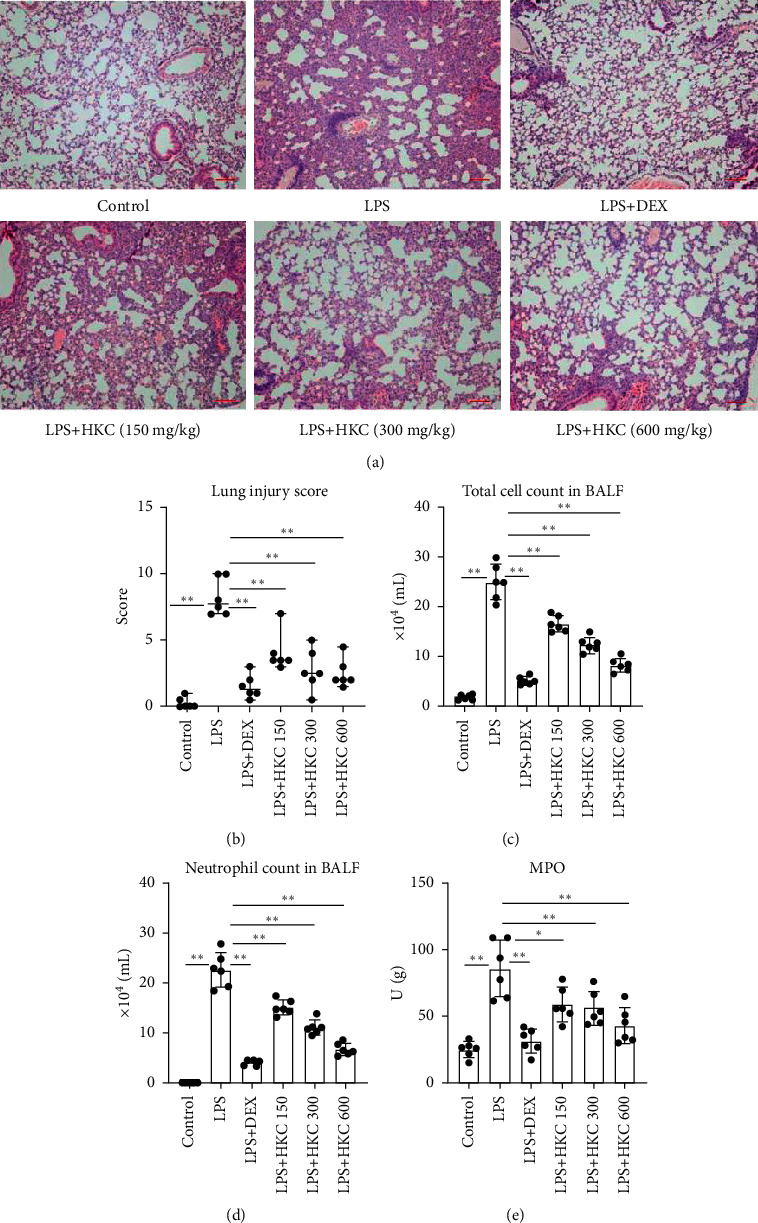
Effect of HKC on the inflammatory response in lung tissues. Histopathological changes in the lung tissues (100x, H&E staining). Mice were intragastrically administered HKC once daily for 5 days. DEX (5 mg/kg) was intraperitoneally injected once 30 min before LPS instillation. Lungs were processed for histological changes (a) 24 h after LPS challenge. Lung injury scores (b) were evaluated. BALF was collected 24 h after LPS instillation to analyse the total cell count (c) and neutrophil count (d). Lung homogenates were prepared 24 h after LPS exposure, and the activity of MPO (e) was determined. Data represent means ± SD (*n* = 6). ^*∗*^*P* < 0.05. ^*∗∗*^*P* < 0.01.

First, lung histopathologic changes and inflammation scores were measured to evaluate the protective effect of HKC on LPS-induced ALI in mice. As shown in Figures 2(a) and 2(b), in control mice, no obvious infiltration of inflammatory cells was observed in the interstitial tissue or airway (score: 0.25). By contrast, the lung tissues of mice in the LPS group exhibited lung oedema and severe neutrophil infiltration (score: 8.25). HKC or DEX administration significantly alleviated these histological changes induced by LPS, characterised with slight interstitial oedema and fewer neutrophils in the interstitium and alveolar spaces than in the LPS group (score for the LPS + HKC group 600 : 2.5, *P* < 0.01, *F* value: 31.67).

Next, the effect of HKC on the recruitment of leukocytes in BALF was investigated. As presented in Figures 2(c) and 2(d), the total cell count and neutrophil count were significantly higher in the LPS group than in the control group (*P* < 0.01). Compared with LPS-challenged mice, HKC- or DEX-treated mice had significantly reduced total cell count (*P* < 0.01, *F* value: 124.5) and neutrophil count in BALF (*P* < 0.01, *F* value: 130.4).

The MPO activity reveals the degree of neutrophil infiltration. In this study, we assessed the MPO activity in the lung tissues. As shown in Figure 2(e), the lung MPO activity in the LPS group was distinctly higher than that in the control group (*P* < 0.01). However, treatment with HKC suppressed this activity (*P* < 0.05 or *P* < 0.01, *F* value: 16.02).

Thus, these results indicated that HKC could suppress the inflammatory response in the lung tissues, especially neutrophil infiltration.

### 3.2. HKC Reduced Plasma IL-6 and BALF TNF-*α* and IL-6 Levels

**Figure 3:**
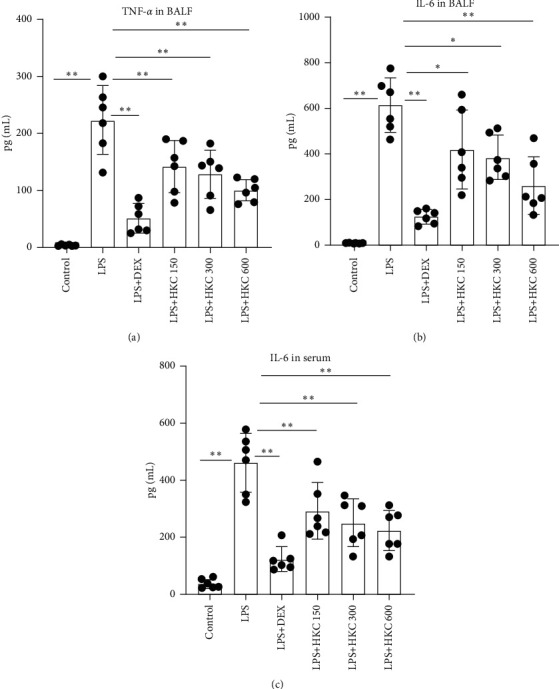
Effect of HKC on the levels of IL-6 in plasma and TNF-*α* and IL-6 in BALF. Mice were intragastrically administered HKC once daily for 5 days. DEX (5 mg/kg) was intraperitoneally injected once 30 min before LPS instillation. The plasma and BALF were collected 24 h after LPS application. The levels of TNF-*α* (a) and IL-6 (b) in BALF and IL-6 (c) in plasma were determined using ELISA. Data represent means ± SD (*n* = 6). ^*∗*^*P* < 0.05. ^*∗∗*^*P* < 0.01.

TNF-*α* and IL-6, as critical inflammatory cytokines, contributed to inflammatory responses during LPS-induced ALI. Compared with control instillation, LPS instillation resulted in an increase in the levels of IL-6 in the plasma and the production of TNF-*α* and IL-6 in BALF (Figure 3). However, HKC or DEX efficiently inhibited the plasma IL-6 level (*P* < 0.01, *F* value: 22.20) and TNF-*α* (*P* < 0.01, *F* value: 24.52) and IL-6 production (*P* < 0.05 or *P* < 0.01, *F* value: 24.09) in BALF.

### 3.3. HKC Increased CAT and GPx Activities in Lung Tissues

**Figure 4:**
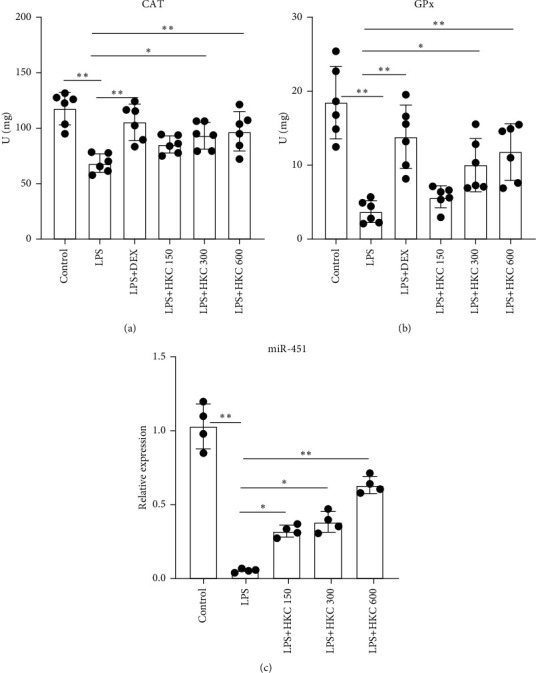
Effects of HKC on the activities of CAT and GPx and the expression of miR-451 in the lung tissues. Mice were intragastrically administered HKC once daily for 5 days. DEX (5 mg/kg) was intraperitoneally injected once 30 min before LPS instillation. Activities of CAT (a) and GPx (b) were determined (*n* = 6). The expression of miR-451 in the lung tissues was determined (*n* = 4). Data represent means ± SD. ^*∗*^*P* < 0.05. ^*∗∗*^*P* < 0.01.

CAT and GPx activities are the indicators of oxidative stress in ALI. Lung CAT and GPx activities were significantly decreased in the LPS group and were markedly higher in the HKC or DEX group than in the LPS group (Figures 4(a) and 4(b), *P* < 0.05 or *P* < 0.01, *F* values: 9.571 and 14.28).

### 3.4. HKC Upregulated the Expression of miR-451 in Lung Tissues

As shown in Figure 4(c), LPS reduced the expression of miR-451 in the lung tissues. However, HKC administration upregulated the expression of miR-451 (*P* < 0.05 or *P* < 0.01, *F* value: 44.54).

### 3.5. HKC Suppressed Nitric Oxide, TNF-*α*, IL-6, and ROS Production in LPS-Stimulated Macrophages

**Figure 5:**
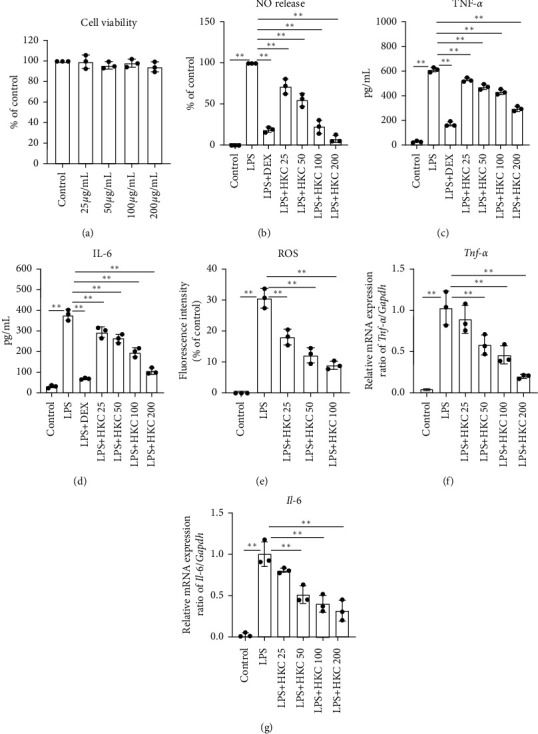
Effects of HKC on nitric oxide, TNF-*α*, IL-6, and ROS production in LPS-stimulated RAW 264.7 cells. Cells were incubated with different concentrations of HKC for 24 h and the cell viability (a) was measured. Cells were incubated with different concentrations of HKC and LPS (100 ng/mL). After 24 h incubation with LPS, culture supernatants were collected for nitric oxide (b), TNF-*α* (c), and IL-6 (d) assays. Intracellular ROS (e) was performed using DCFH-DA. Cells were preincubated with HKC for 2 h and then exposed to LPS for 4 h The expression of TNF-*α* (f) and IL-6 (g) mRNAs was determined through qPCR. Data represent means ± SD (*n* = 3). ^*∗*^*P* < 0.05. ^*∗∗*^*P* < 0.01.

**Figure 6:**
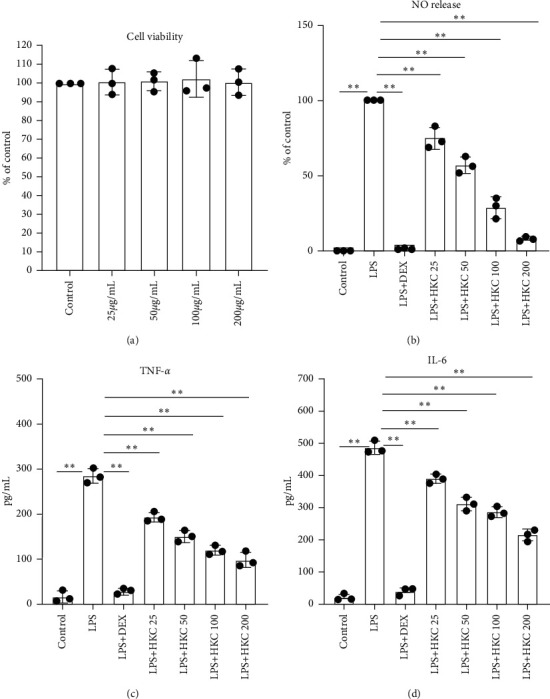
Effects of HKC on nitric oxide, TNF-*α*, and IL-6 production in LPS-stimulated primary peritoneal macrophages. Cells were incubated with different concentrations of HKC for 24 h and the cell viability (a) was measured. Cells were incubated with different concentrations of HKC and LPS (100 ng/mL). After 24 h incubation with LPS, culture supernatants were collected for nitric oxide (b), TNF-*α* (c), and IL-6 (d) assays. Data represent means ± SD (*n* = 3). ^*∗*^*P* < 0.05. ^*∗∗*^*P* < 0.01.

We further performed in vitro experiments to illustrate the effect of HKC on LPS-induced inflammatory responses in RAW 264.7 and primary peritoneal macrophages. HKC did not affect RAW 264.7 cell viability up to a concentration of 200 *μ*g/mL (Figure 5(a)). LPS treatment reduced the levels of nitric oxide, TNF-*α*, IL-6, and ROS, which were reversed by HKC or DEX treatment, compared to those without LPS treatment (Figures 5(b)–5(e), *P* < 0.01, *F* values: 121.6, 378.2, 136.6, and 80.87). LPS-stimulated primary peritoneal macrophages showed results similar to those for RAW 264.7 cells (Figure 6, *P* < 0.05 or *P* < 0.01, *F* value: 242.0, 154.9, and 350.5 for nitric oxide, TNF-*α*, and IL-6 levels, respectively). HKC consistently downregulated LPS-induced transcription of TNF*-α* (*P* < 0.05 or *P* < 0.01, *F* value: 27.55) and IL-6 mRNAs (*P* < 0.01, *F* value: 35.83) in a dose-dependent manner (Figures 5(f) and 5(g)).

## 4. Discussion

The pulmonary innate immune response during ALI is initiated with the activation of residential innate immune cells (such as epithelial cells, alveolar macrophages, and innate lymphoid cells), inducing neutrophil infiltration into the lungs [21]. Multiple inflammatory mediators are released from the activated immune cells, ultimately resulting in neutrophilic inflammation, interstitial oedema, lung parenchymal cell damage, and histohypoxia. Our results demonstrated that HKC effectively attenuated the LPS-induced pulmonary oedema, infiltration of neutrophils, overproduction of pro-inflammatory cytokines, and oxidative stress.

Lung neutrophilic inflammation is a common feature of ALI. Increased expression of neutrophil-related genes has been reported in patients with early stage ARDS [22]. ALI exacerbations are associated with increased neutrophil counts in BALF, and neutrophil-depleted animals show mild histopathological changes in the lung tissues [23]. In the present study, LPS intratracheal administration increased the total cell and neutrophil counts in BALF. The lungs of LPS-challenged mice exhibited a high neutrophil count, along with high MPO activity. Neutrophil infiltration increased the permeability of the alveolar-capillary barrier, subsequently leading to lung oedema [24]. Mice with LPS-induced ALI showed increased lung interstitial oedema. We found that HKC attenuated the symptoms of ALI, including the recruitment of neutrophils in the lung tissues, pulmonary oedema, and histopathological changes.

Alveolar macrophages are the first to encounter incoming pathogens during initiation of the innate immune response in the lung [25]. LPS-induced macrophage activation results in the migration of neutrophils into the lungs. LPS binds to the Toll-like receptor 4 (TLR4) on the membrane of macrophages and recruited MyD88 and activates transcription factors such as nuclear factor-*κ*B (NF-*κ*B), which initiates the transcription and expression of proinflammatory genes, including TNF-*α* and IL-6 [26]. TNF-*α* and IL-6, primarily produced by macrophages and monocytes, are the most promising molecular biomarkers for predicting prognosis [27]. TNF-*α* is a neutrophil chemoattractant that promotes inflammation by upregulating adhesion molecules in leukocyte migration [28]. However, IL-6 has previously been shown to induce rapid (2–6 h) mobilisation of neutrophils from the marginated to the circulating pools in animal models [29]. Pretreatment with HKC dose-dependently reduced the concentrations of TNF-*α* and IL-6 in the BALF of mice with ALI. Similarly, in LPS-induced RAW 264.7 cells and primary peritoneal macrophages, the production of TNF-*α* and IL-6 was inhibited by HKC. These results revealed that HKC has a significant anti-inflammatory effect on LPS-induced mice with ALI.

A clinical study has shown that ARDS is associated with an increase in oxidative stress markers [30]. Excessive ROS generated by the injured endothelium/epithelium as well as recruited leukocytes plays a major role in ALI; increased ROS production can regulate the expression of endothelial cell adhesion molecules via NF-*κ*B signalling [31]. ROS also facilitate the release of TNF-*α* from LPS-induced macrophages [32]. The excessive ROS production observed in ALI can trigger key enzymatic alterations mediated by CAT and GPx, leading to an antioxidant-oxidant imbalance in the lungs. Hydrogen peroxide is reduced to water via enzymatic activities of CAT and GPx. In addition, nitric oxide mediates vasorelaxation. Furthermore, iNOS-mediated increase in nitric oxide production from activated macrophages leads to the reaction of nitric oxide with superoxide free radicals to form a highly oxidative, reactive nitrogen species, peroxynitrite, which can severely damage cellular proteins and DNA [31]. In our study, mice with ALI had low antioxidase activities; however, HKC treatment increased the activities of lung CAT and GPx in mice with LPS-induced ALI, as well as reduced the ROS level and nitric oxide release from LPS-induced RAW 264.7 cells, indicating that HKC significantly inhibited LPS-induced oxidative stress.

Previous studies have verified the potential function of miR-451 in the progression of inflammation-related diseases or oxidative injuries. In neuroinflammation, miR-451 overexpression antagonises LPS-induced microglial activation and reduces the release of IL-6 and TNF-*α* by targeting TLR-4 [33]. miR-451 overexpression suppresses neutrophil migration in response to chemokines [34]. miR-451 provides ischaemic preconditioning-mediated cardioprotection by downregulating the Rac-1/ROS pathway [35]. In addition, miR-451 protects erythrocytes against oxidant stress through the 14-3-3*ζ*/FoxO_3_ pathway, which positively regulates CAT and GPx activities in erythroid cells [36]. Additionally, previous animal studies showed that the ablation of miR-144/451 gene in mice increased the production of IL-6, TNF-*α*, and nitric oxide, as well as the severity of LPS-induced ALI. Moreover, miR-451 overexpression inhibited proinflammatory mediator secretion in LPS-stimulated RAW 264.7 cells (unpublished data). These reports revealed that miR-451 could protect against inflammatory and oxidative responses. In our study, we found that the expression of miR-451 was dramatically decreased in the lungs of mice with ALI. However, the expression of miR-451 was upregulated after HKC treatment, suggesting that the upregulation of miR-451 could be a potential mechanism underlying the anti-inflammatory and antioxidant effects of HKC.

In our study, HKC was orally administered to mice. Based on the clinically recommended dose of HKC (7.5 g/day), a dose of 0.125 g/kg/day is recommended for an adult weighing 60 kg. For rats with glomerular injury, a maximum of 2 g/kg/day of HKC can be administered for 4 weeks [13]. These reports demonstrated that HKC is well tolerated by humans and animals. Additionally, HKC has undergone a strict quantity control procedure as a standardised medicine, and its known bioactive components, including rutin, hyperoside, and isoquercitrin, exhibit high stability [11] with reported anti-inflammatory activity [37–39]. These components mainly contributed to the effects of HKC in LPS-induced ALI in mice.

## 5. Conclusions

Our data demonstrate the anti-inflammatory and antioxidative properties of HKC in LPS-induced ALI in mice and RAW 264.7 cells. The protective effect of HKC against ALI may be associated with the inhibition of LPS-induced inflammatory cytokine production and elevation of antioxidative enzyme activities and miR-451 expression in the lung, resulting in alleviation of LPS-induced inflammatory cell infiltration and oxidative stress. We believe that the present study will help to expand the clinical application of HKC in inflammatory lung diseases.

## Data Availability

The datasets used and/or analysed in the current study are available from the corresponding author upon request.
